# Role of Private Practice Dietitians in Working With People With Mental Illness: A Constructivist‐Interpretivist Qualitative Interview Study

**DOI:** 10.1111/jhn.70349

**Published:** 2026-09-22

**Authors:** Michelle Shwu Huey Hsu, Francis Guo, William Morrow, Andrew Watkins, Scott B. Teasdale

**Affiliations:** ^1^ Keeping the Body in Mind Program, Community Mental Health Services South Eastern Sydney Local Health District Sydney New South Wales Australia; ^2^ School of Life and Environmental Sciences, Faculty of Science University of Sydney Camperdown New South Wales Australia; ^3^ Mindgardens Neuroscience Network Randwick New South Wales Australia; ^4^ Nutrition, Exercise & Social Equity (NExuS) Research Group, Discipline of Psychiatry and Mental Health, School of Clinical Medicine UNSW Sydney Kensington New South Wales Australia

**Keywords:** dietitians, mental illness, private practice, public healthcare, qualitative research

## Abstract

**Aims:**

This study aimed to explore dietitians' views, experiences, and perceived barriers and enablers to working with people with mental illness in Australian private and public mental health settings, with a focus on clarifying the contribution of private practice dietitians to mental healthcare.

**Methods:**

A combination of convenience and snowball sampling was used to recruit dietitians who primarily work in private practice or public mental health sectors. Virtual semi‐structured interviews were conducted in two discrete 3–6‐month blocks between 2021 and 2024. A constructivist‐interpretivist approach informed the development of the interview guide, data collection and analysis processes. Semi‐structured interviews explored experiences, facilitators and barriers of private practice dietitians working with people with mental illness. Data were analysed by three authors using reflexive thematic analysis using semantic, inductive coding.

**Results:**

In total, 14 dietitians completed interviews, comprising 7 working in private practice settings and 7 in public mental health services. Data adequacy was reached with interviews generating 5 main themes and 15 subthemes. Themes included (1) the complex needs of people living with mental illness, (2) access barriers and enablers to private practice dietitian services, (3) gaps in mental health‐specific training and support for dietetics, (4) structural limitations of the private healthcare systems and (5) advantages of integrated care in specialised mental healthcare settings for working with people with mental illness.

**Conclusions:**

To harness the full potential of private practice dietitians in mental healthcare requires targeted reforms to funding, training and referral systems to ensure equitable, community‐based nutrition support.

## Practitioner Points

- Private practice dietitians voice a need for more training and report inherent structural barriers which are limiting their capacity to work effectively with people with mental illness.
- Ease of communication with multidisciplinary team and opportunities for flexible and creative dietetic practice were some advantages of dietitian positions within public mental health services.
- Future advocacy work should address system‐wide training and education gaps, referral and funding models to improve accessibility and integration of high‐quality nutrition care for people with mental illness.

## 1 Introduction

People living with mental illness face a range of diet‐related health challenges that extend beyond their mental health diagnoses [1, 2, 3, 4, 5], and contribute to a 12–16‐year gap in life expectancy compared to the general population [6]. Despite increased recognition of these physical health disparities, rates of obesity, diabetes and cardiovascular disease continue to occur at 1.4–2.0 times higher than the general population [7], and account for 80% of premature deaths in this population [8]. In addition, current treatments for mental illness—such as psychotherapy and pharmacotherapy—are important to reduce symptoms and improve quality of life, but are often only partially effective [9]. As demand for mental health supports continues to rise [10], there is growing interest in holistic, multidisciplinary approaches that include dietary and lifestyle interventions, particularly for conditions such as mood disorders [11, 12].

Evidence supports the effectiveness and cost‐efficiency of dietitian‐led interventions in improving physical health [13, 14] and mental health outcomes, particularly symptoms of depression for people with mental illness [12, 15]. The role of dietitians in the care for people with mental illness has been well described [16, 17]; however, treatment gaps persist.

Australia has a mixed private–public health model, including public hospital services, private health insurance and various government‐funded programmes [18]. The Chronic Disease Management Plan [19], under Australia's universal health care scheme (Medicare) and issued by general practitioners, allows people with chronic or complex physical health conditions to access five subsidised or fully covered (‘bulk‐billed’) sessions with eligible private allied health services including dietitians. The National Disability Insurance Scheme (NDIS) [20], is a separate government programme for people living with significant disabilities to access needs‐based supports and services, including private dietitian services. Private practice dietitians represent over one‐third of Australia's dietetic workforce and are well positioned to provide accessible, community‐based nutrition care [21]. Given that nearly half of all Australians experience mental health concerns during their lifetime [10], and many do not access public mental health services, private practice may be a critical access point for nutrition support. For those that do access public mental health services, dietetic support remains limited [17]. However, there is little exploration on the perspectives and experiences of private practice dietitians in supporting people with mental illness which could help inform critical elements such as training or competency recommendations, and referral pathways.

This study aims to explore the perspectives of dietitians working in private practice and public mental health services in Australia, with a focus on clarifying and optimising the contribution of private practice dietitians to mental healthcare.

## 2 Materials and Methods

This qualitative study was informed by a constructivist‐interpretivist paradigm, acknowledging that dietitians' experiences of working with people with mental illness are socially and contextually constructed. Semi‐structured interviews were used to explore perceived barriers and facilitators within clinical practice, allowing knowledge to be co‐created through researcher–participant interactions and interpretations. Data were analysed using reflexive thematic analysis [22], allowing researchers to explore how participant experiences and perspectives were shaped by the contexts in which they practice. The approach also supported researcher reflexivity and collaborative interpretation, enhancing the depth and credibility of the findings [22]. The study is reported in accordance with the Standards for Reporting Qualitative Research [23].

Recruited participants were dietitians working in Australia in either: (a) private practice settings, or (b) public mental healthcare settings. Dietitians working in public settings with a role specific and limited to eating disorders were excluded. This was to ensure that interviews captured perspectives reflecting mental health conditions more broadly; acknowledging that eating disorders are a specialty of practice in dietetics independent of other mental health conditions [24].

Included mental illness diagnoses were schizophrenia spectrum and other psychotic disorders, bipolar and related disorders, depressive disorders, anxiety disorders, and obsessive‐compulsive and related disorders, as defined in the American Psychiatric Association, Diagnostic and Statistical Manual of Mental Disorders Fifth Edition [25]. Other diagnoses (e.g., eating disorders, dementia, intellectual or developmental disabilities) were excluded. In the context of participants working with both eligible and ineligible mental health diagnoses, focus remained on the eligible diagnoses [26].

**Figure 1:**
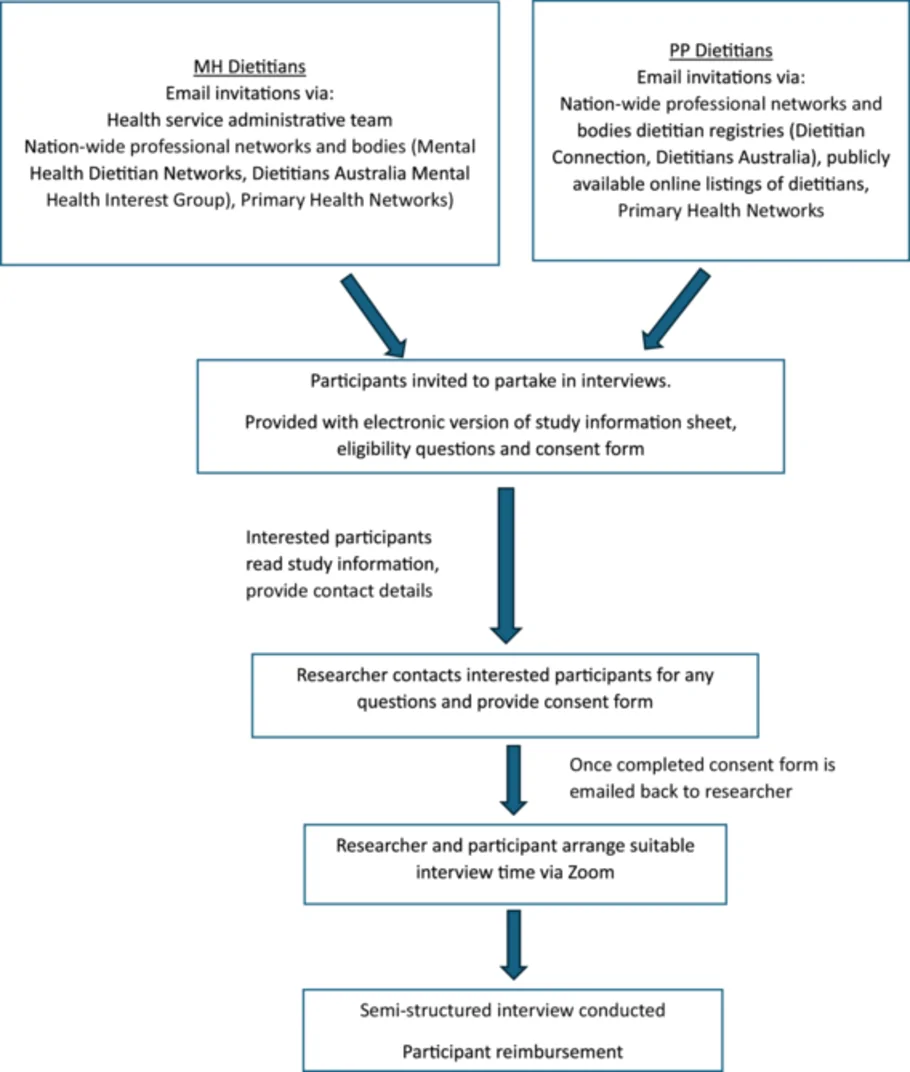
Recruitment flowchart. MH = mental health, PP = private practice. Contact lists of email invitations only included recipients already consenting to receiving profession‐related communications.

Dietitians were recruited via convenience sampling (emails through health service administrative staff, professional mental health dietitian network groups, professional body (Dietitians Australia) and professional education and networking organisation (Dietitian Connection)) and snowball sampling (Figure 1). Recruitment and data collection occurred in two 3–6‐month blocks between 2021 and 2024, due to an extended period of leave and changes in researcher availability. Interested participants were provided with a Participant Information Sheet and provided written informed consent prior to interview.

The study aimed to interview a minimum of 14 dietitians (7 private practice dietitians and 7 public mental healthcare dietitians). Sample size was based on our study aims, specificity of participant groups, quality of interview data and adequacy of data to answer the research question as well as previous studies with similar cohorts and topics [27, 28, 29, 30, 31, 32, 33]. Data adequacy was reached when later interviews contributed no substantively new subthemes and when the overall thematic structure remained stable across participants from both sectors. If data adequacy was not reached, more participants were recruited.

Interviews were conducted as online video calls (Zoom Cloud Meetings (Zoom) platform). Interviews were up to 60‐min in length and were audio‐recorded in‐app. Audio recordings were transcribed verbatim by MSHH and de‐identified for data analysis. Transcripts were saved on secure servers only accessible to the study team. Participants were compensated for their time with a $50 shopping e‐voucher upon completion of the interview. A semi‐structured interview guide (Supplement S1) was used. The interview phase was an iterative and reflexive process—data collection and data analysis were conducted concurrently such that emerging themes from previous interviews helped to guide future interviews [34].

Interviews were conducted by a final year Master of Nutrition and Dietetics research student (FG) and a researcher (MSHH) employed as a mental health dietitian with experience in the public mental health sector and private practice and in qualitative interviewing and methodology. The Master student was in their final year of clinical dietetic training and did not undertake any clinical or teaching activities with any of the participants. The student was also trained in qualitative interviewing principles by research team members (AW, ST), who are experienced in qualitative research, prior to commencing any interviews.

Data were analysed using Braun and Clarke's reflexive thematic analysis [35], adopting an inductive, semantic coding approach. This involved (1) familiarisation with the data, (2) generating codes, (3) searching for themes, (4) consolidating themes, (5) defining themes and (6) production of finalised themes and subthemes [35]. Data were analysed by three authors (MSHH, AW, WM) to achieve richer interpretations and greater rigour. MSHH brought experience from private practice and public mental health sectors, AW and WM are mental health nurses in public mental health sectors and offered interpretations from non‐dietetic lenses. Authors familiarised themselves with the data by independently conducting multiple read‐throughs, notetaking, then collaboratively generating codes from common features across transcripts. These codes were collated to determine key themes. Themes were reviewed for homogeneity within each and heterogeneity between them, collapsing similar themes into broader themes, creating a hierarchy of themes and subthemes. Themes were defined and finalised with supporting quotes to illustrate their meaning. Any discrepancies were reconciled through further meetings until consensus was reached. Throughout analysis, the authors engaged in ongoing reflexive discussions, acknowledging that their clinical backgrounds, training and shared interest in improving nutrition care for people with mental illness can serve as analytic resources to inform interpretation and meaning‐making rather than using coder agreement as a marker of reliability.

This study was approved by South Eastern Sydney Local Health District Human Research Ethics Committee (2021/ETH00987).

## 3 Results

In total, 14 dietitians completed interviews (see Table 1 for demographic and experience details). Majority identified as female (93%), were based in NSW (79%) and worked in metropolitan or major city areas (64%). Participants' career experience ranged from 12 months to over 30 years, with half having less than 5 years' experience. For the mental health dietitians, the majority worked in mental health outpatient or community settings. Several participants had experience across both sectors, offering valuable cross‐contextual insights (see Table 1).

**Table 1:** Demographic characteristics of participants.

Characteristic	Total sample (*n* = 14)	Private practice (*n* = 7)	Mental health (*n* = 7)
Female	13 (93%)	6	7
State			
NSW	11 (79%)	5	6
Victoria	2 (14%)	1	1
Queensland	1 (7%)	1	0
Location of practice			
Metropolitan (major city)	9 (64%)	5	4
Rural	5 (36%)	2	3
Remote	0 (0%)	0	0
Dietetic experience			
≤ 5 years	7 (50%)	3	4
6–15 years	5 (36%)	3	2
15+ years	2 (14%)	1	1
Experience across both sectors	4 (29%)	1	3

Data adequacy was achieved, and analysis generated 5 overarching themes and 15 subthemes. A description of the themes, list of associated subthemes and substantive supporting quotes are presented in Table 2. Narrative for subthemes is presented below.

**Table 2:** Summary of generated themes, associated subthemes and supporting quotes.

Theme	Description	Subthemes	Supporting quotes
1. Perceived needs of people living with MI	Participants described key considerations that guided their dietetic practice when working with people with MI and complex psychosocial backgrounds. Dietitians emphasised the importance of person‐centred, trauma‐informed care and described the need for flexible, intensive support tailored to the clients' readiness and mental health journey. Examples of individualised care in practice included greater flexibility in appointments and cancellations, more frequent sessions over longer timeframes, understanding that behaviour change can occur more gradually. Clients' readiness for nutrition and lifestyle changes as well as their financial capacity to access dietetic services were also acknowledged.	1.1 Shared values of person‐centred and trauma‐informed dietetic practice	‘…unfortunately, the stigma still exists. You know, if there's a a diagnosis written down of Schizophrenia or borderline personality disorder. And there's, you know, trauma history on top of that, or there's substance use on top of that or there's, you know, significant financial constraints’ (Participant 305, MH dietitian). ‘making sure that you're meeting them at their point of need that it's working on issues or goals that are important and meaningful to them. You know, I think all those sorts of considerations that you take in terms of being trauma informed, and recovery orientated’ (Participant 303, MH dietitian)
		1.2 Intensity and flexibility of dietetic care	‘a lot of flexibility… and maybe a little bit more padding’ (Participant 303, MH dietitian) ‘the attendance rate like it's the same in community mental health, you know it is poor attendance. That's why you do, you know, really need to have that close contact to try and get the follow up happening, but yeah, it's I suppose it's always going to be a barrier’ (Participant 135, PP Dietitian) ‘the more sessions that you can have with someone with the history of mental ill health, I think the more effective you can be’. (Participant 301, PP dietitian) ‘sessions are really up to the client themselves. So if they choose to do an online session, we do online. If they prefer a house visit, it'll be travelling to the house […] or maybe at like a coffee shop or something like that if they're, you know, a hoarder or something in the house, just sort of isn't safe to sit in. […] Some people prefer phone call. They don't want to switch their videos on as well. I've had a few clients who we're scheduled to have sort of a video call and they get quite overwhelmed when I log in and will straight away turn our cameras off and just change it to a phone consult’. (Participant 302, PP dietitian)
		1.3 Readiness of clients with mental illness to engage in dietetic intervention	‘a little bit harder versus say someone that didn't have a background or a history of mental ill health and they, you know, had just been recently diagnosed with type 2 diabetes then from my point of view 3 sessions is kind of enough to give them the nutrition education, I can see some improvements […] I feel a bit more confident in being able to be an effective dietitian’ (Participant 301, PP dietitian) ‘because of the mental health conditions like depression, anxiety or whatever like if they are having one of those bad days, one of those bad days happen to be one of the days that they see me that I feel is really hard to motivate them’ (Participant 121, PP dietitian) ‘patients with mental health conditions, they are unemployed or they have a very unstable career. […] less financial support to, you know, buy food or even like fund their medications so food safety then becomes another barrier. […] and then the nutrition becomes the last one on the priority list’. (Participant 121, PP dietitian) ‘I think often we do see that people living with mental illness can be from a lower socioeconomic but I also just think like the nature of the illness involves a lot of medical costs.[…] there's only so many psychiatrists and psychologist and GP subsidies that exist so people are already outlaying a lot of money to help with specifically just the mental health side of things […] when it comes down to it, a lot of people rightly so would probably prioritize seeing the psychiatrist over the dietitian’ (Participant 71, MH dietitian)
		1.4 Working creatively to support people with MI in different stages of their MH journey	‘I guess that's when client centered care becomes so important in that, OK, there's no guidelines for working with people with OCD. But what's important for that person? What's important for their physical health and what's important for their mental health and so if you take that view, then there will be evidence to support reaching goals, whether that's around the relationship with food, whether that's around cardiometabolic health, whether that's around gut health, I guess, yeah, you've got to look at the whole person, not just their mental illness and their whole physical and mental health’ (Participant 305, MH dietitian) ‘when they're in hospital, they're unwell and that's not really the time for lifestyle education. So the place for lifestyle education is when they're at their wellest in the community. So I think that is the place where community dietitians, in our district are private dietitians, have the ability to make that life‐changing lifestyle education and help people with their home diets as well, which is their normal and their long term, whereas we're seeing things and often adjusting to a hospital diet which is not a normal diet for them’ (Participant 308, MH dietitian)
2. Access considerations to PP Dietetic services	Participants described how access to PP dietitian services is limited by poor knowledge of the role PP dietitians play in the physical health needs of people with MI as well as referral pathways. Even then, dietetic interventions within subsidised frameworks are heavily limited by their financial viability for PP dietitians.	2.1 Awareness of the role of dietitians in MH by clients, clinicians and other dietitians	[Educating clients about their role and] ‘who the heck am I and why should they spend their time with me’. (Participant 71, MH dietitian) ‘I don't think independent of prompting and a lot of support that these people would access a private practice dietitian. I don't think a lot of people, if they haven't come across (public healthcare system) would be really aware of that dietitians do play a role in mental healthcare and I think a lot of people probably wouldn't even know that dietitians exist’. (Participant 71, MH dietitian) ‘I have a handful of GP's that refer… Have one GP that – this is in private practice – that like refers me I swear every single client (laughs). But I love too, that with our relationship and our letters and my communication, she now will, like, send me everything I need or like the way she words her referrals is even different now, which is kind of cool. And I feel like it's nice to see that advocacy or education come through’ (Participant 304, PP dietitian, prior public MH sector experience) ‘I think I have looked at it and the hit rate isn't high. I think there's no one that really specifically targets mental health, but there's a few, like larger clinics that have, you know, mental health listed under a plethora of other topics that they can help with’. (Participant 71, MH dietitian)
		2.2 Limitations of the bulk billing system for effective work with people with MI	‘when you've only got a client for 20 minutes. It's very much OK. What's what can I do for you? What? What's the issue? Here's some information… Good luck Godspeed (laugh). You know, whereas if you've got, you know, like you've got 20 visits. It's like, OK, well, let's just cruise through this a little bit’. (Participant 300, PP dietitian) ‘all my appointments is around 20 minutes to be in line with the Medicare requirement and that's often not enough for someone with mental illness. […] if I remember correctly that you know the Medicare has commenced the the item number for people with eating disorders […] they don't have anything else or other and other mental health illnesses.’ (Participants 121, PP dietitian) ‘financially is huge, like a lot of people are on different financial support and or potentially aren't in a place where they can work, yeah. It's, I would say not common for people to be financially in a position where they're willing to pay for private dietetics, unfortunately […] I often don't offer it because I just know it's not gonna be considered so we look at other options instead’ (Participant 308, MH dietitian) ‘I guess the other thing with youth too […] a lot of it is prevention rather than treatment, so to get the CDMP you kind of need to have diabetes or hypertension and a lot of our young people don't have that, […] like the culture around you know, at the moment it seems very treatment based rather than that prevention’ (Participant 71, MH dietitian).
3. MH‐specific dietetic knowledge and training for PP dietitians	Participants described the lack of widespread MH‐specific training and education for dietitians in both their university degree and beyond. It was said to be common for Dietitians to assume that MH dietetics involved eating disorders as opposed to the expansive work in nutrition support for people living with other psychiatric diagnoses and psychosocial challenges. Prioritising MH‐specific learning relied upon personal interest rather than systemic supports. Participants shared a belief that Dietetic professional bodies could support them by promoting the scope of dietitians' role in MH spaces.	3.1 Lack of MH‐specific training and supports for students and working dietitians	‘there's one lecture on mental illness. If they get placed at our service, or maybe one or two other services, then they will get some placement experience. But most of them will get placed in general community dietetics […] So I guess people can go through their whole degree and really only have one hour learning […] the dietetic complexities of working with people with mental illness’. (Participant 305, MH dietitian) ‘I just remember feeling very undertrained, very underprepared and completely unawares of how to deal with any of this situation’ (Participant 302, PP dietitian) ‘I think there's a lot of misconception in terms of what mental health dietetics even is for dietitians so you know, often people do think ohh eating disorder or gut health’ (Participant 304, PP dietitian, prior public MH sector experience) ‘I guess the other thing would be for CPD to be focused on mental illness as much as it's focused on other topics. […] a lot of CPD focus is on cardiometabolic diseases or gastrointestinal sort of focus, but not a lot focuses specifically on mental health, which means that I guess people are not feeling confident building skills to work appropriately with this population group’. (Participant 305, MH dietitian)
		3.2 MH training depends heavily on individual dietitians' interest in MH	‘a skill that you kind of learn on the job’ (Participant 300, PP dietitian) ‘I think my clients are my greatest teachers, to be honest, no doubt about it’ (Participant 302, PP dietitian) ‘working in private practice, you're not in that team. You don't have supervision, you don't have the CPD opportunities coming up. So I definitely must have to seek that out myself […] but now in [public health MH setting], you know, we, we luckily have all the supervision we need so that's really good’ (Participant 135, PP dietitian) ‘it's difficult from private practice because also you're a generalist most of the time, you don't have a lot of opportunity to do that CPD in mental health. Like you opt to do diabetes or fertility, or bariatric, or paediatrics, where you're more likely going to – that's your higher number of referrals’. (Participant 306, MH dietitian)
		3.3 PP dietitians want and need more MH‐specific training and support	‘it probably does come down to more targeted kind of like CPD and training opportunities and skill development because I think it's like without the knowledge or awareness, you are doing the best that you can with the knowledge and tools and skills that you have. Because you aren't aware of what. You don't know what you don't know’ (Participant 304, PP dietitian, prior public MH sector experience) ‘it's probably something that dietitians or the DA need to push more on. And make that more accessible for dietitians… like I wouldn't even know where to go to improve my education around dietetics in mental health. I mean, I'm sure that I'm sure there's stuff out there, and to be honest, I've never even thought before this to delve into it. So that's an issue in itself, isn't it?’ (Participant 300, PP dietitian) ‘I think having, like even though I am a contractor and I'm not directly engaged with other employees, the support from the management team is incredible, incredible, like especially being a mobile service, […] if I have trouble contacting people or difficult, a really difficult client and I just need advice, I can call any of them and they will help me straight away. […] a company that didn't specialize in disability or NDIS dietetics for a new graduate to come in, you wouldn't have that same level of support and understanding’. (Participant 154, PP dietitian) ‘we do have our Dietitian Mental Health Network, but I don't know how [PP Dietitians] would know about that to get in contact with it. As a newer dietitian, it wouldn't have been something that I would have been aware of so it would have to come from some other source’. (Participant 308, MH dietitian)
4. Inherent structures in the PP healthcare system that limit dietetic service delivery for people with MI	The nature of PP—including business constraints, administrative burden and siloed care—misaligned with the intensity and continuity of care required for people with MI. Both MH and PP dietitians described the current private healthcare system and siloing nature of PP business posed limitations to holistic care for clients. For people living with MI, administrative requirements can be higher than clients without MI due to the complexities of support networks involved in their care.	4.1 Nature of running a dietetic business in the PP ecosystem	‘you book an appointment and they don't show up, or they've been in hospital, so you have to cancel last minute and in order for the business to run successfully, it's something that you need to bill. But it's that balance of me billing this and maintaining the integrity of business is then you missing out or cutting an hour or two hours off a very limited amount of time that we had anyway. […] NDIS or the CDM perhaps doesn't take into account those sorts of access issues that might occur.’ (Participant 303 MH dietitian, prior PP experience) ‘One hour for an initial assessment, 4 hours left to cover 12 months, that's, for someone that has significant food trauma, 4‐hour appointments, it's not going to make a dent really. Like it's not, it's not going to be intensive enough that you're going to make a lot of ground. So, going about things with their other stakeholders that are likely to have more contact because they might have more funding for those stakeholders is so important to making sure that you're getting the whole team involved in this main goal’ (Participant 154, PP dietitian) ‘I haven't really done any marketing or I've kind of maybe just sent out like different emails or, you know, hey, like new to the area or like, practicing, hey, I'm a dietitian, these are the areas I work in but haven't like spelled out’ (Participant 304, PP dietitian, prior public MH sector experience)
		4.2 Siloing nature of PP dietetic work and opportunities for connection and communication between sectors	‘it's just me in an office, […] Like that would be like really good to work in the same building as say, you know, psychologist. […] rather than, you know, typing up a letter or writing up an e‐mail, you can just see them or have like a weekly catch up, you know, weekly meeting and talking about the patients that you share like that would be that would be pretty cool’. (Participant 301, PP dietitian) ‘there's sort of limited possibilities of soft handovers for those that are really quite complex and needing that. […] it's just a bit of a disservice to both the person and then that clinician that it's a bit rinse and repeat of starting fresh’. (Participant 303, MH dietitian) ‘we do MDT and we always ask the psychologist in the private practice, you know, a social worker we've had, we've had social workers who are in NGOs call in. We just don't have that many here but we've never had an MDT with the private practice dieticians’ (Participant 306, MH dietitian)
		4.3 Current PP healthcare system does not cater for the intensity of dietetic care for people with MI	‘You're not gonna have a very good experience if you go in and just hit the ground running with your dietetics assessment straight away. They're not going to want to engage and I think for us engagement is really challenging and we have to work on building rapport and understanding where the person's are in their life and meeting them there […] otherwise on their next potential admission or next community appointment they're, “I don't wanna see a dietitian”’ (Participant 308, MH dietitian) ‘I would love to have limitless time with them, so ideally like an NDIS sort of home care package style funding where you know there's no, it's not a sprint to get to the solution, you can kind of go along with them on the journey. […] those periods where things are going well and everything is great, and then life happens and it derails them and it would be nice to be able to see a client through all those different stages to provide different levels of support and education throughout those stages’. (Participant 300, PP dietitian)
5. Advantages of specialised MH settings for working with people with MI	MH dietitian participants had unique opportunities to work closely with MH specialised healthcare providers in a way that better met the needs of people with MI. MH dietitian roles in MH specialised settings were salary‐based positions in public healthcare systems such as hospitals or outpatient clinics and community centres. This allowed dietitians to have longer‐term therapeutic relationships and opportunities for creative, non‐traditional dietetic practice.	5.1 Ease of communication with the care team in MH settings	‘You know who the care coordinator is, they might have other standing appointments within the service that you can coordinate. They might be coming in for a depot where you can just drop in and say hey and they might be coming in for olanzapine group. And so I guess there's an increased opportunity for that to cross pathways, which I can imagine would be very limited in a private setting’. (Participant 303, MH dietitian) ‘I have daily contact with doctors, daily contact with social workers, daily contact with psychologists. They're informing your work in terms of what the latest is’ (Participant 306, MH dietitian)
		5.2 MH specialised settings allow for longer bouts of clinical care and therapeutic relationships	‘when you do invest a lot of time and resources and energy into building that rapport and that relationship […] when you do start to see results, it is really, really rewarding. So I think yeah, that's… you can see that benefit not only on their physical health, but also the effect on their mental health journey’. (Participant 71, MH dietitian) ‘I guess it gives us the luxury of working at a slower pace and taking it at the rate that the person would like to go at because you know, if they need to come in once a week, then they can. You know, they're not out of pocket $100 each time’ (Participant 305, MH dietitian) ‘there is a lot more scope for flexibility with appointments in terms of times and frequency when you're not having to be accountable in terms of billing as such, I know that working with the NDIS provider and sort of being accountable in terms of meeting billables per week, you really had to do a lot of forecasting in terms of the amount of appointments that you would try to fit in to make sure that that was happening’. (Participant 303, MH dietitian)
		5.3 MH specialised settings enable creative non‐traditional dietetic practice to meet the needs of people with MI	‘nutrition education to counselling, behavioural change, motivational interviewing, goal setting or more practical things like meal planning, meal preparation, shopping, to a supermarket tours, practical one‐on‐one sessions in the kitchen, and then also not currently, but historically have offered like more group settings’ (Participant 71, MH dietitian) ‘I guess historically it's been an extremely isolating role for a lot of people to work in. […] just being it's really sort of created a safe space in terms of being able to ask questions and sort of try to understand what's happening outside of your service or your scope and I think that's something that is incredibly hard as a private practice dietitian in terms of not having that network of people to go to or ask questions about or just explore’ (Participant 303, MH dietitian)

Abbreviations: MH = mental health, MI = mental illness,PP = private practice.

### Theme 1: Perceived Needs of People Living With Mental Illness

#### Subtheme 1.1—Shared Values of Person‐Centred and Trauma‐Informed Dietetic Practice

Both private practice and mental health dietitians acknowledged the challenges faced by many people living with mental illness including trauma, psychosocial disadvantage and stigma. In this context, participants described the importance of working in a person‐centred, trauma‐informed and recovery‐oriented approach.

#### Subtheme 1.2—Intensity and Flexibility of Dietetic Care

Dietitians described providing flexible cancellation processes and more follow‐up contact to accommodate the higher cancellation and no‐show rates compared to clients not living with mental illness, influenced by fluctuating mental health symptoms, psychosocial stressors and competing priorities. More frequent sessions were favoured, understanding that building rapport can take time to create a safe therapeutic alliance for nutritional interventions.

#### Subtheme 1.3—Readiness of Clients With Mental Illness to Engage in Dietetic Intervention

Psychosocial disadvantages can prevent people with mental illness from accessing dietitian services, attending sessions and being ready to incorporate dietary changes into their lives. Financial capacity of clients with mental illness to afford private practice dietitian services was discussed by all participants as an independent and significant barrier.

#### Subtheme 1.4—Working Creatively to Support People With Mental Illness, in Different Stages of Their Mental Health Journey

Dietitians valued working creatively and innovatively to adapt traditional nutrition practices to better meet the complex psychosocial and mental health presentations of people with mental illness. Mental health dietitians diversified nutrition support into cooking groups, supermarket trips and involving the multidisciplinary team in nutrition goals. Private practice dietitians harnessed carers and support networks to improve engagement and to support adherence to nutrition plans outside of dietetic appointments. Private practice dietitians had an important role in nutrition support during phases of one's mental health journey when they are more mentally well.

### Theme 2: Access Considerations to Private Practice Dietetics Services

#### Subtheme 2.1—Awareness of the Role of Dietitians in Mental Health by Clients, Clinicians and Other Dietitians

Educating clients about their role was an important part of initial sessions. Similarly, private practice dietitians described building connections with referring health professionals as opportunities to improve knowledge of their role in mental health referrals. Mental health dietitians described challenges in identifying local private practice dietitians to refer to and supporting clients to follow through with private practice dietitian referrals when discharging from public mental health services.

#### Subtheme 2.2—Limitations of the Bulk Billing System for Effective Work With People With Mental Illness

All participants spoke of the limitations of the Medicare system and financial constraints. Dietitians described the gap in Medicare subsidies for people with mental illness to access private practice dietitians. Out‐of‐pocket charges were a significant deterrent for mental health dietitians considering private practice services as a discharge pathway when clients transition away from public mental health services to primary healthcare.

### Theme 3: Private Practice Dietetics for Mental Health Specific Dietetic Knowledge and Training Gaps

#### Subtheme 3.1—Lack of Mental Health Specific Training and Supports for Dietitian Students and Working Dietitians

All participants described receiving minimal mental health training in their dietetic university degree. Participants wanted more mental health related content in university programmes and as working private practice dietitians. Benefits included improving the standard of mental health dietetics knowledge and skillset across graduating dietitians.

#### Subtheme 3.2—Mental Health Training Depends Heavily on Individual Dietitians' Interest in Mental Health

Private practice dietitian participants described self‐directed learning on the job and seeking mental health specific training and supervision independently. Other nutrition topics may be prioritised over mental health due to cost return of referrals for conditions such as diabetes and heart disease compared to mental health.

#### Subtheme 3.3—Private Practice Dietitians Want and Need More Mental Health Specific Training and Support

Private practice dietitian participants wanted more training and upskilling in mental health dietetics through training courses and supervision. Specialised support may be available for private practice dietitians who work under larger business models but this was not always the case.

### Theme 4: Inherent Structures in the Private Practice Healthcare System That Limits Dietetic Service Delivery to People Living With Mental Illness

#### Subtheme 4.1—Nature of Running a Dietitian Business in the Private Practice Ecosystem

Private practice dietitians held in‐depth knowledge of the private practice ecosystem and how to work within the constraints of NDIS and Medicare billing frameworks. When working with people with mental illness there was additional administrative workload and unpaid hours for complex care coordination. Self‐promotion and advocacy for more mental health dietetic referrals became lower priority.

#### Subtheme 4.2—Siloing Nature of Private Practice Dietitian Work and Opportunities for Connection and Communication Between Sectors

Private practice dietitians described the siloing nature of their work and barriers to easily liaise with other clinicians involved in client care. When individuals are discharged from public services, transitioning to private practice services is usually constrained by affordability and funding, however, these were opportunities to improve thoughtful handover and continuity of care for clients.

#### Subtheme 4.3—Current Private Practice Healthcare System Does Not Cater for the Intensity of Dietetic Care for People With Mental Illness

Private practice dietitians described the constraints of the private practice system that limited their ability to meet the intensity of care people with mental illness benefit from. Follow‐up contact cannot be as frequent as they want to improve attendance and goal attainment, and their business cannot handle as many cancellations while Medicare schemes can only subsidise limited sessions to do the work required in the intervention.

### Theme 5: Advantages of Specialised Mental Health Settings for Working With People With Mental Illness

#### Subtheme 5.1—Ease of Communication With the Care Team in Mental Health Settings

Mental health dietitians described advantages of being integrated into multidisciplinary mental health teams, including the ability to have ad hoc communications and timely collaborative care planning, and access to mental health training and supervision.

#### Subtheme 5.2—Specialised Mental Health Settings Allow for Longer Bouts of Clinical Care and Therapeutic Relationships

Mental health dietitians described the advantages of specialised mental health settings for building therapeutic relationships with clients with mental illness. Physical spaces were trauma‐informed and salary‐based roles accommodated more intense service provision and flexible care.

#### Subtheme 5.3—Specialised Mental Health Settings Enable Creative Non‐Traditional Dietetic Practice to Meet the Needs of People With Mental Illness

Mental health dietitians described how working in specialised mental health settings allowed them to work creatively with people with mental illness to make nutrition support accessible and engaging. Mental health dietitians also spoke about the value of connecting and networking with other mental health dietitians across other mental health services. Peer support and cross‐pollination of ideas amongst colleagues helped them navigate the non‐traditional landscape of mental health dietetics.

## 4 Discussion

This study explored the views across the continuum of dietetic care (private and public services) to explore the potential of private practice dietitians to support people with mental illness. The perspectives highlighted that many of the identified barriers and enablers extend across sectors involved in mental healthcare. Participants highlighted the importance of person‐centred, flexible and intensive care, including frequent sessions aligned with a person's mental health journey. Private practice participants described the tension between delivering gold‐standard care and maintaining a financially sustainable private practice business navigating limited subsidised pathways, administrative burden and gaps in mental health‐specific training. Challenges relating to funding structures, accessing mental health‐specific training and integration within multidisciplinary care systems were consistently described across both sectors. These findings suggest that efforts to improve dietetic service provision for people with mental illness should be considered at a system level, with coordinated strategies that support workforce development, service integration and continuity of care across public and private settings.

Participants described desirable conditions for delivering effective care within mental health services, including being integrated into multidisciplinary mental health teams, flexible service models, streamlined communication and access to peer networks. However, the integration of dietitians into mental health services continues to evolve with many services remaining under‐resourced and/or with a limited scope of practice [36]. Further, many people with mental illness do not engage with public mental health services and instead present to primary care. Given that private practice dietitians make up over a third of Australia's dietetic workforce [21], and that nearly one in two Australian's experience a mental health condition in their lifetime [10], optimising the private practice sector's capacity to support people with mental illness represents a significant opportunity. These findings also highlight the challenges and opportunities associated with transitions between public mental health services and community‐based care, including private practice dietitians. This is consistent with stepped‐care and recovery‐oriented models of mental health service delivery.

The central role of person‐centred, trauma‐informed care identified in this study reflects broader literature advocating for these approaches in mental health and chronic disease management [31, 37, 38, 39]. Participants described using their clinical judgement and counselling skills to deliver evidence‐based care while fostering strong therapeutic relationships—a hallmark of effective dietetic practice [28, 29, 40]. However, these principles were challenged by systemic pressures. The balance between ethical, high‐quality care and financial sustainability echoes findings from other studies, where practitioners cited tension between clinical values and business constraints [37, 41, 42, 43].

A key barrier identified was the misalignment between the needs of people with mental illness and how current dietetic service models are structured, particularly in private practice. This disconnect is most evident in the limitations of existing funding mechanisms. Australia's primary subsidy scheme for allied health, the Chronic Disease Management Plan [19], was consistently identified as inadequate for people with mental illness due to its low session cap (total of five sessions per year allocated across any mixture of allied health professions), often needing a co‐payment (similar to private health insurance), administrative demands and misalignment with best‐practice guidelines for chronic conditions [42, 43, 44]. For instance, the Chronic Disease Management Plan fails to accommodate clinically impactful long‐term dietary interventions for conditions like diabetes and hyperlipidaemia [45, 46], which is also more prevalent in people with mental illness. Other schemes such as the NDIS [20]—Australia's funding programme for eligible people with disabilities to support their health, wellbeing and ability to live more independently—offer more appropriate funding structures; however, access to funding plans and adequate funding within them for dietetic intervention is challenging [47], and participants noted that administrative tasks and higher cancellation rates reduce the effectiveness of allocated time.

Participants also highlighted the importance of enhancing mental health dietetic training through university curricula and professional development. As growing numbers of graduating dietitians entering private practice landscape, existing feelings of being unprepared and under‐supported in their roles can compound when working with clients with mental illness [48, 49, 50]. As a sector that is highly likely to work with clients with mental illness, strengthening mental health competencies and supervision will improve workforce readiness [48, 49, 50, 51]. Many participants relied on self‐directed learning, which was influenced by individual capacity and interest. There was a perceived scarcity of private practice dietitians promoting themselves as skilled in mental health nutrition, which may reflect broader issues of confidence and role clarity. Increasing awareness among general practitioners and referring health professionals about the role of dietitians in mental illness could facilitate more referrals and integrated care [52, 53].

This study should be interpreted with the following limitations. First, the small sample size and demographics impact the generalisability of findings across Australia. This sample size was small relative to each sector (mental health dietitians and private practice dietitians), were predominantly from NSW and from metropolitan or regional locations. Remote regions of dietetic practice were not represented in this study, despite the well‐recognised disparities in workforce and resource distribution that can impact nutrition care in these settings. Several subthemes were supported by multiple participants across both sectors, reinforcing their relevance despite the relatively small sample size. The relatively large number of subthemes in relation to the sample size reflects the exploratory, multi‐level focus of the analysis, rather than over‐fragmentation of participant accounts. While we judged data to be adequate for developing the reported themes, recruitment approaches may have preferentially attracted dietitians with greater interest or experience in mental health, limiting the applicability to those with less experience. Further, the distribution strategy meant we could not determine the reach of advertisement. Second, the extended recruitment period may have impacted findings, acknowledging that private practice and mental health contexts can evolve over time. However, themes and subthemes remained strongly consistent across interviews and recruitment timepoints, especially regarding funding limitations, administrative burden, gaps in education and training. Importantly, during the study period (2021–2024), there were no substantial changes identified to key funding schemes relevant to dietetic services (e.g., Medicare Chronic Disease Management Plan, NDIS). Third, mental health dietitians primarily drew experience from working with serious mental illnesses (schizophrenia, bipolar disorder and major depressive disorder), whereas private practice dietitians more commonly described supporting high‐prevalence mental illnesses (depression and anxiety). Despite this, generated themes and subthemes were appropriate across the spectrum of mental disorders. Fourth, the interview guide was designed iteratively based on experience and existing literature and organically elicited experiences and perspectives without constraining responses within predefined implementation domains. Future studies can consider applying implementation frameworks to examine barriers and facilitators more systematically. Finally, due to the nature of professional networks, researchers may have been known to participants. We aimed to minimise this influence through supervision, debriefs and training by two investigators experienced in qualitative research.

## 5 Conclusion

Private practice dietitians are a scalable yet underutilised workforce that can complement public mental health services in delivering community‐based nutrition care for people with mental illness. However, structural, training and funding barriers limit the sector's capacity to meet the intensity and integration required for working with this population. Expanding public mental health dietetic positions alongside improving training, funding models, referral pathways and collaboration across sectors is essential for more equitable access to integrated nutrition care.

## Author Contributions

Conceptualisation: Michelle Shwu Huey Hsu. Data curation: Michelle Shwu Huey Hsu and Francis Guo. Formal analysis: Michelle Shwu Huey Hsu, Andrew Watkins, William Morrow and Francis Guo. Funding acquisition: Scott B. Teasdale. Investigation: Michelle Shwu Huey Hsu and Francis Guo. Methodology: Michelle Shwu Huey Hsu, Scott B. Teasdale and Andrew Watkins. Project administration: Michelle Shwu Huey Hsu. Resources: Scott B. Teasdale. Software: N/A. Supervision: Scott B. Teasdale, Andrew Watkins and Michelle Shwu Huey Hsu. Validation: all authors. Visualisation: N/A. Writing – original draft preparation: Michelle Shwu Huey Hsu. Writing – review and editing: all authors.

## Disclosure

All authors agree with the manuscript and declare that the content has not been published elsewhere.

## Ethics Statement

Subject to review and approval by the Human Research Ethics Committee.

## Consent

Interested participants were provided with a Participant Information Sheet and provided written informed consent prior to interview.

## Conflicts of Interest

The authors declare no conflicts of interest.

## Supporting information


Supporting File

## Data Availability

The data that support the findings of this study are available on request from the corresponding author. The data are not publicly available due to privacy or ethical restrictions.
