# Supplementary material for: Role of Private Practice Dietitians in Working With People With Mental Illness: A Constructivist‐Interpretivist Qualitative Interview Study
Source: J Hum Nutr Diet. 2026 Sep 22;39(5):e70349. doi: 10.1111/jhn.70349 (PMC13598358; doi:10.1111/jhn.70349)
Supplement: Supplementary file 1 [file JHN-39-0-s001.docx]

Supplement 1. Semi-structured interview guide, informed by a constructivist-interpretivist approach and designed iteratively based on experience and existing literature.

Interview Guide

[Interview conducted through Zoom Cloud Meetings platform which enables audio-only recording]

Introduction

Thank you very much for your time and agreeing to speak with me about your views on private practice (PP) dietitians. Your help and participation means a lot to us, and it is an important contribution to our research - we really appreciate your time.

Before we start, I’ll quickly explain what we are going to do. This interview should take about 60 minutes, give or take a few minutes. In this interview, I want to learn about your experiences and your opinions about PP dietitians working with people living with mental illness (MI). For the purpose of this interview and research study, when we say “people with MI”, we are referring to psychiatric diagnoses including schizophrenia spectrum and other psychotic disorders, bipolar and related disorders, depressive disorders, anxiety disorders, and obsessive-compulsive and related disorders as listed in the American Psychiatric Association, Diagnostic and Statistical Manual of Mental Disorders fifth edition (DSM-5). In this interview, we are not referring to any other diagnoses in the DSM-5 such as eating disorders and dementia, intellectual or developmental disabilities, however if you have worked with someone with an eligible MI who also lives with these other conditions we can include these in the interview and our questions will still be focused on the eligible diagnoses.

I’ll be asking you questions such as what experiences you have had with clients with MI in the past, whether you would want to work with client with MI in the future, and the factors that make it easier or challenging to work with them. So I am really interested in your experiences, your opinions and your ideas. While the word ‘interview’ may sound intimidating, please remember that there are no wrong or right answers, it is just what you personally experienced.

All the information that you share with us is completely confidential and your participation is voluntary. For data analysis purposes, we would like to record the audio of this interview only although you do have the option of not recording. Regarding confidentiality, once the recording starts I will introduce you with your participant number and refrain from using your name to protect your identity and your audio recording will be destroyed once it is transcribed into a typed version. This way, there is no way for anyone to be able to identify you through the interview data. Do you give permission for me to record the interview?

[If the participant provides verbal consent to recording] I will now click the Record button to make sure that it catches what we are saying.

If you do not wish to answer any particular question I ask you have the right to skip over it or come back to it later in the interview. There is no obligation to answer any of all questions. You may also ask to stop immediately.

I may write a few memos throughout the interview, this is just to remind me about something which I want to ask you later on. Please feel free to ask me to stop or repeat anything at any time.

Is there anything that you would like to ask me before we start?

Warm up

To put this interview into context, let’s start with some background about you.

Can you please tell me about:

- What made you decide to take part in this interview?
- Your current practice situation?
- How long have you been working as a dietitian?

Past experiences working with people with MI

Q1. Have you worked with a client where a MI diagnosis has been disclosed by the person or their carer or via referral in a PP setting?

(If the participant indicates previous experience(s) working with a client with MI)

Probe questions (as appropriate in the conversation):

- What brought the client to see you? / what did you think about seeing the client at that time? / how has that changed now? / how long did you see them for?
- Can you please describe what it was like working with the client with MI? / How did you manage that? / and then what did you do?
- Overall, how was your experience? / What was that like for you? / What made you think that?

Comparison to working with people without MI

- Was your experience with the client with MI different to working with a person not living with MI of similar sociodemographics? / If so, how? / Why do you think that is? / Is there anything else that is different?

Barriers and enablers of working with people with MI

Q2. What did you like the most about working with clients with MI?

Probe questions:

- Why was that so positive for you? / What about working with clients without MI? / Is there anything else?

Q3. Were there any notable challenges/barriers experienced when working with clients with MI?

Probe questions:

- How did this/these affect your consultations/effectiveness as a PP dietitian? / Is there anything else?
- What would have made your sessions more effective and/or efficient? / Why do you think that would have helped?
  - How do financial factors play a part in working with clients with MI?

Expectations for working with people with MI

(Skip to here if participant has nil experience working with a client with MI according to above sections)

Q4. Ideally for you, what would it be like to work with a client with MI?

Probe questions:

- What would you want to get out of working with a client with MI? / Why is that important to you?
- Do you think there is anything a PP dietitian can offer to a client with MI that is different to dietitians in any other setting? / What do you think is unique or special about what a PP dietitian can do for a client with MI? / Why do you think that is? / Why do you think that is valuable?
- Can you please describe what an ideal session would look like with a client with MI? / Why is that important to you? / Anything else?
- What do you think would help make all the above possible?

Wrap up

What would you like done so that PP dietitians can work better with people with MI?

Prompts if re-wording required:

- If you were to work with a client with MI again, how would you like that experience to look?

Is there any else that you would like to tell me about your experience with clients with MI? / Is there anything that I may have missed which you think is important and you would like to tell me?

Lastly, I would like to thank you very much for your time and for sharing your personal experiences with me. It has been wonderful speaking with you and your help in this study is invaluable. Without you, our research would not be possible.

I will now end the recording.

As an appreciation of your time, we will organise for a $50 Westfield voucher to be emailed to you.
